# Activation of Nrf2 in the liver is associated with stress resistance mediated by suppression of the growth hormone-regulated STAT5b transcription factor

**DOI:** 10.1371/journal.pone.0200004

**Published:** 2018-08-16

**Authors:** John Rooney, Keiyu Oshida, Naresh Vasani, Beena Vallanat, Natalia Ryan, Brian N. Chorley, Xuting Wang, Douglas A. Bell, Kai C. Wu, Lauren M. Aleksunes, Curtis D. Klaassen, Thomas W. Kensler, J. Christopher Corton

**Affiliations:** 1 NHEERL, US-EPA, Research Triangle Park, NC, United States of America; 2 Immunity, Inflammation, and Disease Laboratory, National Institute of Environmental Health Sciences, Research Triangle Park, NC, United States of America; 3 University of Kansas Medical Center, Kansas City, KS, United States of America; 4 Rutgers University, Ernest Mario School of Pharmacy, Department of Pharmacology and Toxicology, Piscataway, NJ, United States of America; 5 University of Washington, Seattle, WA, United States of America; 6 Department of Pharmacology & Chemical Biology, School of Medicine, University of Pittsburgh, Pittsburgh, Pennsylvania, United States of America; 7 Department of Environmental Health & Engineering, Johns Hopkins Bloomberg School of Public Health, Baltimore, Maryland, United States of America; University of Nebraska Medical Center, UNITED STATES

## Abstract

The transcription factor Nrf2 (encoded by *Nfe2l2*) induces expression of numerous detoxifying and antioxidant genes in response to oxidative stress. The cytoplasmic protein Keap1 interacts with and represses Nrf2 function. Computational approaches were developed to identify factors that modulate Nrf2 in a mouse liver gene expression compendium. Forty-eight Nrf2 biomarker genes were identified using profiles from the livers of mice in which Nrf2 was activated genetically in Keap1-null mice or chemically by a potent activator of Nrf2 signaling. The rank-based Running Fisher statistical test was used to determine the correlation between the Nrf2 biomarker genes and a test set of 81 profiles with known Nrf2 activation status demonstrating a balanced accuracy of 96%. For a large number of factors examined in the compendium, we found consistent relationships between activation of Nrf2 and feminization of the liver transcriptome through suppression of the male-specific growth hormone (GH)-regulated transcription factor STAT5b. The livers of female mice exhibited higher Nrf2 activation than male mice in untreated or chemical-treated conditions. In male mice, Nrf2 was activated by treatment with ethinyl estradiol, whereas in female mice, Nrf2 was suppressed by treatment with testosterone. Nrf2 was activated in 5 models of disrupted GH signaling containing mutations in *Pit1*, *Prop1*, *Ghrh*, *Ghrhr*, and *Ghr*. Out of 59 chemical treatments that activated Nrf2, 36 exhibited STAT5b suppression in the male liver. The Nrf2-STAT5b coupling was absent in in vitro comparisons of chemical treatments. Treatment of male and female mice with 11 chemicals that induce oxidative stress led to activation of Nrf2 to greater extents in females than males. The enhanced basal and inducible levels of Nrf2 activation in females relative to males provides a molecular explanation for the greater resistance often seen in females vs. males to age-dependent diseases and chemical-induced toxicity.

## Introduction

Oxidative stress reflects an imbalance between reactive oxygen species (ROS) and the ability of a biological system to detoxify reactive intermediates or to repair resulting damage. Disturbances in the normal redox state of cells through the production of ROS can damage critical cellular components leading to injury or disease. Oxidative stress plays roles in chemical-dependent and -independent cytotoxicity and tumor promotion [1, 2]. There are currently ~13 adverse outcome pathways (AOPs) that include oxidative stress as a key or modulating event in pathways that lead to an adverse outcome in animal or human tissues (https://aopwiki.org/; accessed December 22, 2017). Induction of oxidative stress has been observed following exposure to chemicals that activate xenobiotic receptors including the aryl hydrocarbon receptor (AhR), constitutive androstane receptor (CAR), and peroxisome proliferator-activated receptor α (PPARα). Chemicals that activate these receptors cause liver cancer through nongenotoxic mechanisms and generally act at the promotion stage by increasing cell proliferation [3–5].

Cellular oxidants activate the nuclear factor erythroid 2-related factor 2 (NF-E2/related factor 2, Nrf2) transcription factor [6] which is often considered to act as a first line of defense from cellular damage due to unchecked oxidative stress. Nrf2, a basic region-leucine zipper (b-Zip)–type transcription factor heterodimerizes with members of the small Maf transcription factor family. The Nrf2-Maf heterodimers bind to antioxidant response elements (AREs) in the promoters of genes that encode protective enzymes involved in xenobiotic detoxification, antioxidant response, and proteome maintenance [6, 7]. Under conditions of low oxidative stress, Keap1 (Kelch ECH associating protein 1), a cytosolic Nrf2 repressor protein, binds to and marks Nrf2 by polyubiquitination for proteasomal degradation through a complex with Cullin 3 (Cul-3)–based E3 ligase [8]. Increases in ROS or electrophiles trigger Keap1 to undergo a conformational change, resulting in disruption of the complex and abrogation of Nrf2 ubiquitination, allowing for direct translocation of nascent Nrf2 to the nucleus [9]. A growing number of exogenous and endogenous stressors as well as chemopreventative chemicals activate Nrf2, including ROS, reactive nitrogen species, lipid aldehydes, and electrophilic xenobiotics and their metabolites [10, 11]. The Keap1-Nrf2 redox sensing system is highly conserved across vertebrate and invertebrate species making it a critical defense mechanism to cellular stress [12].

Clinical and preclinical studies show sexually dimorphic responses of the liver to various stressors with females often exhibiting resistance to damage compared to males. Examples of heightened susceptibility of males include liver ischemia reperfusion, hemorrhagic shock-resuscitation, liver cirrhosis, and hepatocellular carcinoma. Estrogen has been suggested to be a factor responsible for this sexual dimorphism [13–19]. One estrogen-regulated transcription factor linked to sexual dimorphism and stress responses is signal transducer and activator of transcription 5b (STAT5b), one of seven mammalian STAT transcription factors [20, 21]. Like other family members, STAT5b responds to a variety of extracellular cytokine and growth factor signals, including growth hormone (GH) [22–24]. The pituitary gland secretes GH in a sex-dependent manner under the control of gonadal steroids [25, 26]. In rodentia and to a lesser extent in humans, the pattern of GH secretion differs between the sexes. In rats and mice, plasma GH levels are highly pulsatile in males, in which hormone peaks are followed by well-defined GH-free intervals. Pituitary GH release in females occurs more frequently, resulting in more continuous plasma GH levels [25, 27]. These sex-dependent plasma GH profiles, in turn, regulate liver gene expression at the level of transcription [28]. The sex dependency of most sexually dimorphic genes was lost in the livers of STAT5b-null male mice [29–31]. Regulation of STAT5b through GH secretion, is affected by physiological stimulators (e.g., exercise, nutrition, sleep) and inhibitors (e.g., free fatty acids, glucose) [27], in addition to gonadal steroids [32]. A large number of factors have been shown to disrupt GH signaling and sexually dimorphic gene expression in male mice including chemical exposure; this disruption of STAT5b leads to feminization of the male-specific gene expression pattern [33, 34].

There is a growing body of evidence that Nrf2 is activated in mice with defects in GH signaling, under conditions in which the liver genome would be feminized. These mice have a number of overlapping traits including dwarfism, increased resistance to stressors, and increased longevity [35]. Fibroblasts isolated from Snell *Pit1* dwarf mice have elevated mRNA levels for several Nrf2-dependent genes, as well as cellular traits, such as elevated glutathione levels, resistance to lipid peroxidation, that are known to be modulated by Nrf2 function [36]. A set of Nrf2-dependent mRNAs was elevated in the livers young adult Ames *Prop1* and Snell dwarf mice in the absence of chemical induction [36, 37]. More recently, Ghrh-null mice were shown to have higher nuclear accumulation of Nrf2 protein in hepatocytes compared to wild-type mice and express higher levels of Nrf2 targets [38]. Thus, evidence supports the relationship between genetic disruption of the GH hypothalamic-pituitary-liver axis and Nrf2 activation. However, a comprehensive assessment of the relationships between STAT5b status and Nrf2 activation especially after chemical exposure has not been examined.

In the present study, a gene expression biomarker was used to identify factors in a microarray database that led to Nrf2 activation or suppression. We then determined the relationship between Nrf2 activation and suppression of STAT5b using a previously characterized gene expression biomarker [33].

## Materials and methods

### Overall strategy for identification of factors that affect Nrf2

**Fig 1:**
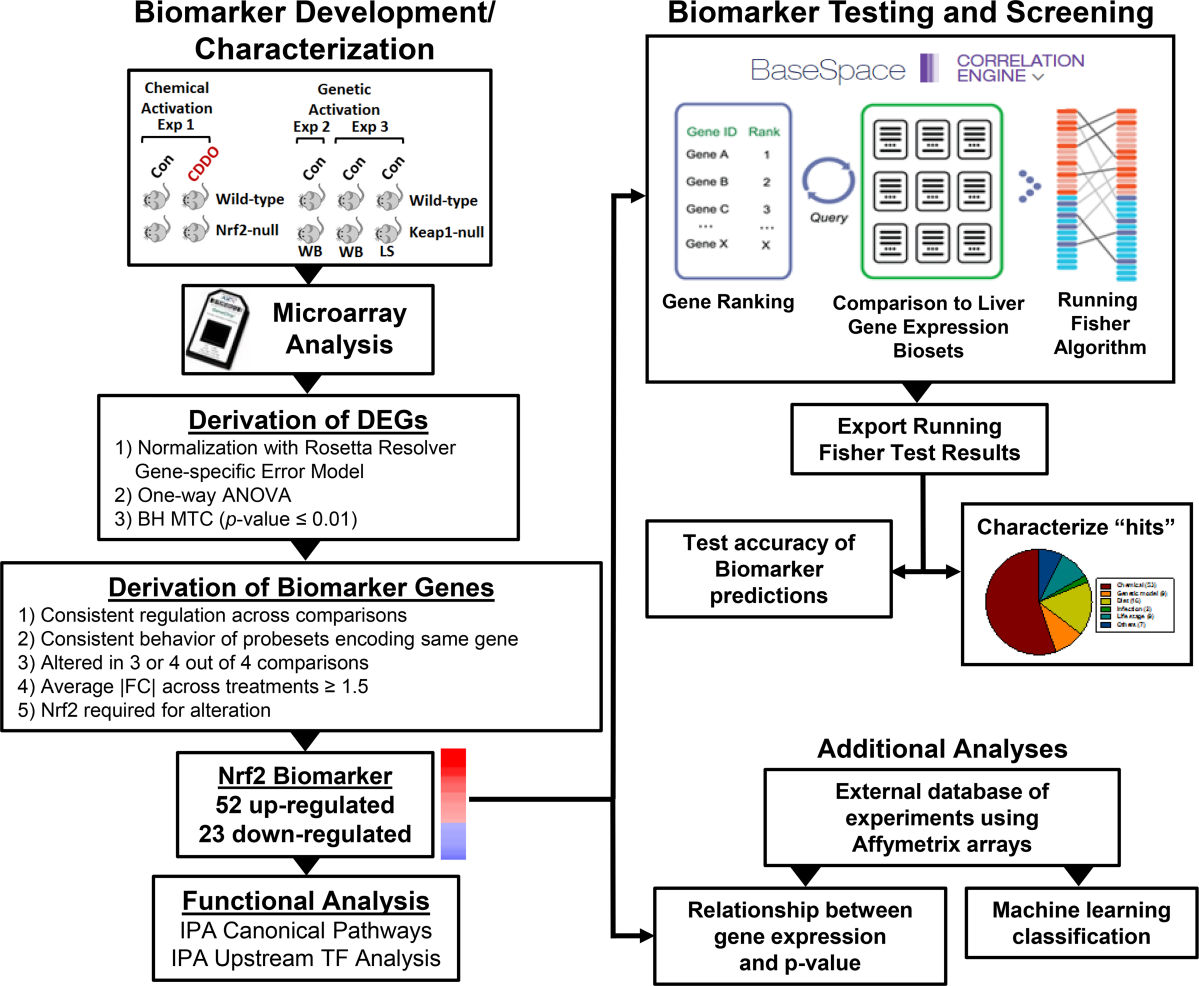
Scheme for Nrf2 biomarker development and characterization for screening of a mouse liver gene expression compendium. Left, biomarker development and characterization. A number of microarray experiments were used to create the Nrf2 biomarker. Wild-type and Nrf2-null mice were treated with CDDO-Im (experiment 1; [47]). Wild-type and Keap1-null (whole body (WB) or liver-specific (LS)) mice were compared in experiments 2 [47] and 3 [49]. Differentially expressed genes (DEGs) were identified using Rosetta Resolver as indicated. Biomarker genes were identified from the DEGs after applying a number of filtering steps. Genes in the biomarker were evaluated by Ingenuity Pathway Analysis (IPA) for canonical pathway enrichment and potential transcription factor regulators. Right, biomarker testing and screening. The Nrf2 biomarker was imported into the BSCE environment in which protocols rank ordered the genes based on their fold-change. Screening was carried out by comparison of the biomarker to each bioset in the BSCE database using a pair-wise rank-based enrichment analysis (the Running Fisher algorithm). The results of the test including the direction of correlation and p-value of the test for each bioset in the compendium were exported and used to populate a master table containing bioset experimental details. A test of the accuracy of the biomarker predictions was carried out with treatments that are known positives and negatives for Nrf2 activation. Screening “hits” were characterized to determine the factors that modulate Nrf2 activation. Additionally, an external gene expression database of experiments performed with Affymetrix arrays was used in a classification analysis as well as to determine the relationship between expression of Nrf2 biomarker genes and p-values from the Running Fisher algorithm. Part of the Figure was adapted from a Figure in [44] and [40].

The methods used in this study are outlined in Fig 1. Evaluation of the effects of different factors on Nrf2 activity required a gene expression biomarker of Nrf2-dependent genes and an annotated database of gene expression profiles of statistically filtered genes (also called biosets). The Nrf2 biomarker is a list of genes with associated fold-change values that reflect average differences in expression after chemical or genetic activation and that require Nrf2 for these gene expression changes. A commercially available gene expression database provided by Illumina (BaseSpace Correlation Engine (BSCE); https://www.illumina.com/products/by-type/informatics-products/basespace-correlation-engine.html; formally NextBio) was used as the starting point to create a compendium of mouse liver biosets. The BSCE database contains over ~21,600 highly curated, publically available, omic-scale studies across 15 species including ~134,000 lists of statistically filtered genes (as of October, 2017). Each list (bioset) is compared to all other biosets in the database using a fold-change rank-based statistical algorithm called the Running Fisher algorithm which allows an assessment of the overlap in genes and whether those genes are regulated in a similar or opposite manner. In this study, only biosets from mouse liver, mouse primary hepatocytes, or hepatocyte-derived cell lines were evaluated. Available information about each bioset was extracted from BSCE and used to populate a compendium of information about the experiments. Each bioset was further annotated using information derived from the original GEO submission and/or the original publication. Each bioset was annotated for the type of factor (e.g., chemical) and the name of the factor (e.g., phenobarbital) examined. To assess Nrf2 activation or suppression, the Nrf2 biomarker was uploaded into the BSCE database and compared to all biosets in the database using the Running Fisher algorithm. Results of the test were exported and used to populate the annotated compendium with p-values of each comparison. We have previously used this analysis strategy to accurately identify factors that activate or suppress other transcription factors (AhR, CAR, PPARα, STAT5b, and estrogen receptor α) [33, 34, 39–42].

### Animal experiments

No animal experiments were conducted as part of this study. However, we did utilize tissues from animals that were part of published studies as discussed below.

### Microarray analysis of livers from oltipraz-treated mice

Gene expression was measured in the livers of wild-type or Nrf2-null mice treated with oltipraz or vehicle in which mice were treated each day for 4 days with 75 mg/kg/day [43]. Four biological replicates were measured for each of the 4 genotype-treatment groups. Gene expression was evaluated using Affymetrix mouse exon arrays (MoEx-1_0-st-v1). Liver RNA was isolated using a modified guanidinium isothiocyanate method (TRIzol®, Invitrogen) and was further purified using silica membrane spin columns (RNeasy®, Qiagen, Valencia, CA). RNA integrity was assessed by the RNA 6000 LabChip® kit using a 2100 Bioanalyzer (Agilent Technologies, Palo Alto, CA). All subsequent procedures of hybridization, washing and scanning were carried out according to the manufacturer's recommendations. A Gene Level Differential Expression Analysis was carried out using Affymetrix Transcriptome Analysis Console v1.0.0.234 using the *Mus musculus* genome version mm9. A total of 23,238 genes were analyzed. Statistically significant genes were identified by one-way ANOVA with a false discovery rate (Benjamini-Hochberg test) of ≤ 0.01. The raw microarray files have been archived in Gene Expression Omnibus (GEO) under accession number GSE85222.

### Identification of differentially expressed genes in BSCE microarray datasets

Almost all of the statistically filtered gene lists used in our study were generated using BSCE standardized microarray analysis pipelines and are available in a searchable annotated format in the BSCE database [44]. Additional gene lists were generated using the methods described below. We had previously shown that the choice of the method to derive the lists of significant genes does not affect the results or conclusions of the study [33].

### Identification of differentially expressed genes in an external database

Independent of the BSCE database, a database of gene expression changes was assembled by our group using comparisons from mouse liver, mouse primary hepatocytes and hepatocyte-derived cell lines. All of these experiments were conducted using Affymetrix microarrays. Affymetrix .cel files were downloaded from publicly available sources including GEO and ArrayExpress, which were first analyzed by Bioconductor SimpleAffy [45] to assess sample quality. The .cel files from individual studies were normalized using Rosetta Resolver® version 7.1 Affymetrix Rosetta-Intensity Profile Builder software (Rosetta Inpharmatics, Kirkland, WA). Statistically significant genes were identified by one-way ANOVA with a false discovery rate (Benjamini-Hochberg test) of ≤ 0.01. A total of ~890 biosets were created and annotated as described below using information from the original study. Master tables were built using the common probe sets shared by Affymetrix 430A or 430_2 chip types. A subset of this data was used to examine the relationships between the p-value from the Running Fisher test and the expression behavior of the genes in the biomarker. All statistically filtered gene lists were uploaded into BSCE after filtering for │fold-change│ ≥ 1.2.

### Annotation of a mouse liver gene expression compendium

Annotation has been described in our previous study [40]. Assignments of sex were made from the GEO or ArrayExpress submission or from the original paper as described earlier [33]. It should be noted that the microarray comparisons in the database are from studies carried out with different strains of mice.

### Machine learning classification analysis

Analyses were performed using BRB-ArrayTools version 4.2.1 Stable Release developed by Dr. Richard Simon and BRB-ArrayTools Development Team (http://linus.nci.nih.gov/BRB-ArrayTools.html) [46]. The methods used here were the same as those described in our previous analysis [40]. Two training sets were used for predicting Nrf2 activation. The first training set consisted of samples from wild-type and Nrf2-null mice and included 18 positives and 18 negatives. The second training set consisted of the same set but lacked the control and treated Nrf2-null samples and included 15 positives and 9 negatives. The derived classifiers of 175 and 92 probesets, respectively were then used to predict Nrf2 activation of the remaining samples. A test set of samples known to be positive or negative for Nrf2 activation (13 and 215 samples positive or negative, respectively) came from a number of studies in which mice or mouse primary hepatocytes had known Nrf2 activation status.

### Construction of a Nrf2-dependent gene expression biomarker

Probe sets that comprise the Nrf2 biomarker were derived from comparisons using livers of wild-type and Nrf2-null mice treated with CDDO-Im for 6 hrs [47] and untreated hypomorphic Keap1-floxed mice (whole body knockdown: Keap1^FA/FA^) [48] or additionally featuring hepatocyte-specific targeting (AlbCre::Keap1^FA/FA^) compared to control wild-type mice [47, 49]. The overall strategy was similar to that reported earlier for AhR, CAR, PPARα, and STAT5b biomarkers [33, 34, 39–41]. The strategy is also analogous to that used by Bild et al. [50] to find genes regulated by oncogenic factors in cancer cell lines. Statistically filtered gene lists from the studies were compared and probe sets which met the following criteria were identified: 1) altered in 3 or 4 out of the 4 comparisons (CDDO-Im treated wild-type, and the three Keap1-null comparisons), 2) exhibited the same direction of change in the lists, 3) the │average fold change│ was ≥ 1.5-fold, 4) probe sets that encoded the same gene had identical direction of change after exposure, 5) were not also statistically altered in the same direction in CDDO-Im treated Nrf2-null mice, and 6) were not also found in the gene expression biomarkers for AhR, CAR, PPARα, and STAT5b [33, 39–41]. The final list of 75 probe sets (which collapsed to 48 genes) and their average fold-changes were uploaded to BSCE. The genes in the biomarker are found in S1 File.

### Classification prediction of Nrf2 function

Biosets from microarray experiments in which the Nrf2 activation state was known were manually curated from the following studies: GSE55001, GSE55084, GSE54597, E-MEXP-1231, E-MEXP-153, E-MEXP-347, E-MEXP-438, GSE10082, GSE10769, GSE1093, GSE11287, GSE15633, GSE15859, GSE16381, GSE16777, GSE20944, GSE23780, GSE24751, GSE25142, GSE3150, GSE33575, GSE34423, GSE35124, GSE39313, GSE40120, GSE40773, GSE4259, GSE6721, GSE867, GSE8969, GSE55002, and GSE55003. The number of biosets used to test for Nrf2 activation was 67 positives and 14 negatives. Unlike more traditional machine learning classification methods, optimal conditions for classification were not derived from gene behavior as the biomarker was fixed. In this and in our previous studies [39–41], the biomarkers were compared to known positives and negatives using the Running Fisher algorithm. Studies with the biomarkers for AhR, CAR, PPARα, and STAT5b showed that a cutoff of Running Fisher algorithm p-value ≤ 10^−4^ after a Benjamini Hochberg correction of α = 0.001 resulted in a balanced accuracy of 88%, 97%, 98%, and 98% for AhR, CAR, PPARα and STAT5b activation, respectively [33, 39–41]. The same cutoff resulted in a balanced accuracy of Nrf2 activation of 96% (described in Results). The entire list of biosets chemical exposure experiments described in the present study that exhibited Nrf2 activation or Nrf2 suppression (p-value ≤ 10^−4^) is found in S1 File.

### Chromatin immunoprecipitation sequencing (ChIP-seq) analysis

ChIP-seq data from experiments that exposed mouse cells to Nrf2-activating chemicals were obtained from GEO (2 biological replicates from CDDO-Im treated C2C12 mouse myoblast cell line [GSM2076703, GSM2076704]; 3 biological replicates from diethyl maleate-treated primary mouse macrophages [GSM1944624, GSM1944626, GSM1944628]). Briefly, raw sequencing reads downloaded from NCBI Sequence Read Archive (SRA) were mapped to the mouse genome (mm9) using the BWA (Burrows-Wheeler Aligner) [51]. Replicates were combined and Nrf2 binding peaks were determined by MACS version 2.0 [52]. For each peak, the nearest gene was called based on the distance from the peak summit (mid-point of peak) to the transcription start site. Genes nearest to Nrf2 peaks previously calculated in two additional studies (murine embryotic fibroblasts isolated from Keap1-null mice and diethyl maleate-treated mouse hepatoma cells) were also used [53, 54]. Genes from all four studies were collated and compared to the Nrf2 biomarker genes.

### Ingenuity pathways analysis

The full list of genes in the Nrf2 biomarker was analyzed using the Ingenuity Pathways Analysis (IPA) canonical pathway and upstream analysis functions. The significance for canonical pathways was calculated using a right-tailed Fisher's Exact test by IPA. The p-value is the probability that the Nrf2 biomarker gene list would coincide with the IPA gene list. Upstream analysis function of potential regulators of Nrf2 biomarker genes was based on the number of differentially expressed downstream genes in the biomarker and quantified using a Z-score and p-value. The Z-score is a correlation measure of how consistent the direction of expression changes in the biomarker gene list matches the direction of change from the annotated literature for targets in the biological or regulatory group.

### Evaluation of selected genes by real-time RT-PCR

Livers used for the RT-PCR experiments were from wild-type and Nrf2-null mice treated with the Nrf2 activator oltipraz (75 mg/kg/day for 4 days) as described above. The levels of expression of selected genes were quantified using real-time reverse transcription-PCR (RT-PCR) analysis. Briefly, total RNA was reverse transcribed with murine leukemia virus reverse transcriptase and oligo(dT) primers. The forward and reverse primers were designed using Primer Express software, version 2.0 (Applied Biosystems, Foster City, CA). The SYBR green DNA PCR kit (Applied Biosystems, Foster City, CA) was used for real-time PCR analysis. The relative differences in expression between groups were expressed using cycle threshold (Ct) values (Pfaffl et al., 2002). The Ct values of the genes were first normalized with β–actin and glyceraldehyde 3-phosphate dehydrogenase (GAPDH) of the same sample. Means and S.D. (n = 3–5) for RT-PCR data were calculated by Student's or Aspin-Welch’s t-test. The level of significance was set at p ≤ 0.05.

### Additional analysis

Regression analyses were carried out in Excel. R^2^ and F-test p-values were determined.

## Results

### Characterization of a Nrf2 biomarker

A number of computational strategies were initially examined to identify Nrf2 modulation in genomic databases. We first examined the utility of machine learning algorithms to identify gene sets that would correctly predict Nrf2 activation status. None of the models were adequate for predicting Nrf2 (described in S2 File).

**Fig 2:**
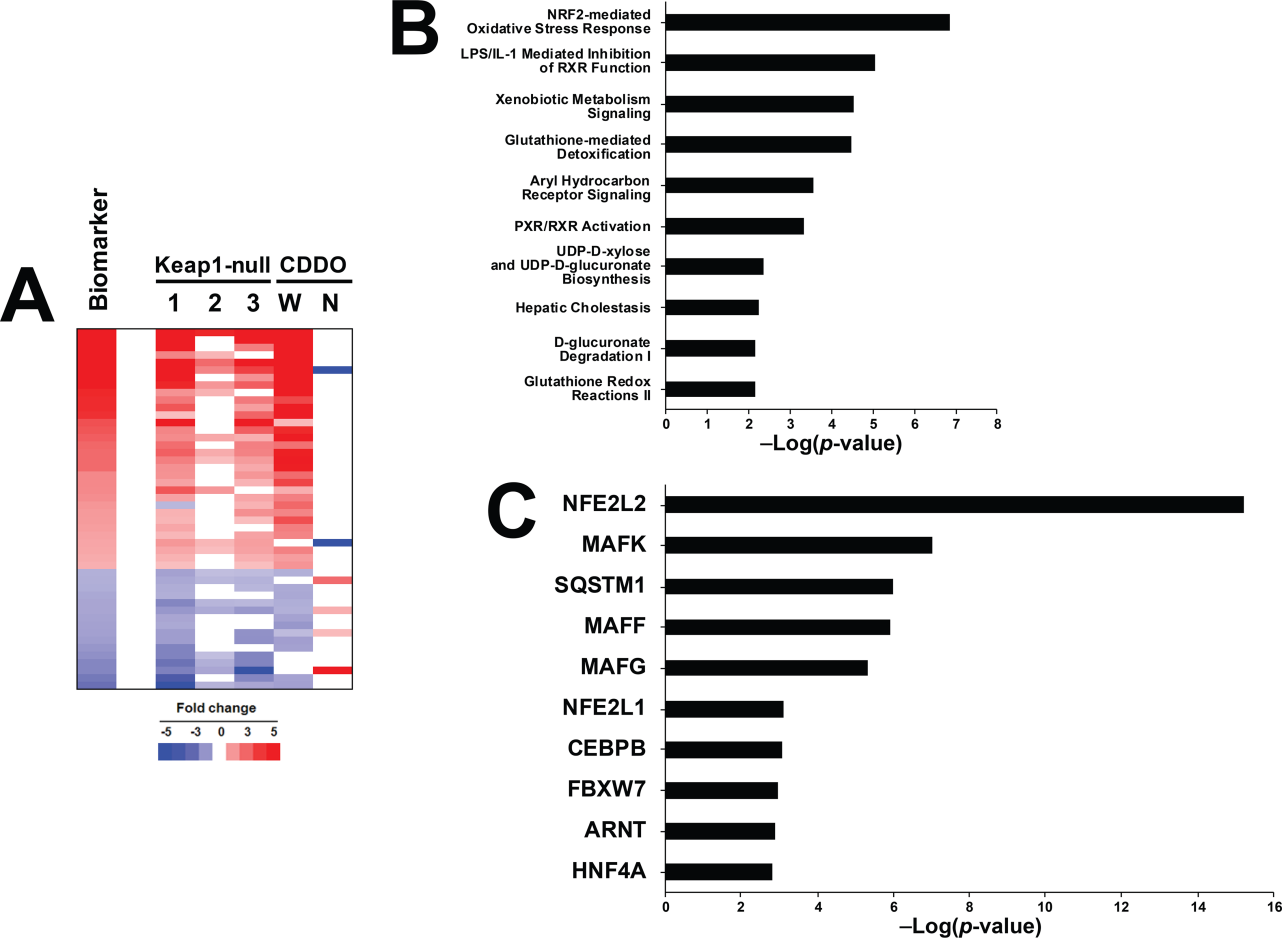
Characterization of a Nrf2 biomarker. A. The Nrf2 biomarker. (Top) Heat map comparing the expression of the genes in the biosets used to create the biomarker to the Nrf2 biomarker itself. Gene expression profiles from the livers of Keap1-null mice compared to corresponding control wild-type mice (1–3) and from the livers of wild-type (W) or Nrf2-null (N) mice exposed to CDDO-Im (CDDO) for 6 hours. Genes that were similarly regulated by genetic or pharmacological activation of Nrf2 were identified as detailed in the Methods. The biomarker represents the average expression of the genes across the biosets that exhibit genetic or pharmacological activation of Nrf2. The Keap1-null vs. wild-type comparisons were 1) hepatocyte-specific-null (GSE55001), 2) whole body-null (GSE55001), and 3) hepatocyte-specific null (GSE15633). B. Significant canonical pathways represented by the genes in the Nrf2 biomarker. Genes were examined by Ingenuity Pathways Analysis. C. Significant factors predicted to act as upstream regulators of the genes in the Nrf2 biomarker as determined by Ingenuity Pathways Analysis.

A rank-based enrichment analysis strategy called the Running Fisher algorithm [44] as evaluated for prediction. To use the algorithm, a Nrf2 biomarker was built as described in the Methods. A total of 75 probe sets (52 with increased expression and 23 with decreased expression, collapsing to 48 genes) were identified which exhibited similar regulation by either pharmacological or genetic activation, and that were not altered in the same direction in Nrf2-null mice by CDDO-Im. Fig 2A shows the consistent expression of the biomarker genes either in the three Keap1-null vs. wild-type mouse comparisons or after CDDO-Im exposure in wild-type mice. Many of the genes are known targets of Nrf2 including *Nqo1*, *Abcc3* and *Ces1*. Notably absent was *Hmox1* which was induced by CDDO-Im in wild-type mice, but not in Keap1-disrupted mice (data not shown).

To evaluate the Nrf2 biomarker genes for direct interactions with Nrf2 (and presumably direct transactivation), genes were compared to existing mouse Nrf2 ChIP-seq datasets. In total, 3641 genes mapped nearest to Nrf2 binding regions (S1 File). Of the 48 biomarker genes, 28 (58.3%) mapped near Nrf2 binding regions in mouse cells as defined by these datasets (Chi-squared test with Yates correction p<0.0001, indicating these 28 biomarker genes were also Nrf2 ChIP genes not by chance). Although the available ChIP-seq databases did not use hepatocyte cells, the combination of the different types cell lines assessed can give a general, overlapping Nrf2-bound gene signature that is likely to be active, given that the universal oxidative stress response is active in many cell types. Given this, we recognize there may be hepatocyte-specific gene targets for Nrf2 that are not represented in this analysis and therefore may have missed some genes in the biomarker that are direct targets for Nrf2 transactivation in hepatocytes. The overall evidence supports the statement that the majority of the biomarker genes are likely direct targets of the Nrf2 transcription factor.

Nrf2 biomarker genes were evaluated for canonical pathway enrichment by Ingenuity Pathway Analysis (IPA) (Fig 2B). The top 10 pathways enriched with the biomarker genes included those previously associated with Nrf2 regulation (“Nrf2-mediated Oxidative Stress Response”, “Glutathione-mediated Detoxification”, “Glutathione Redox Reactions II”). The upstream analysis function of IPA identified transcription factors that regulate the biomarker genes (Fig 2C). The top scoring transcription factor was *Nfe2l2* (Nrf2) (p-value ~1E-15). Other transcription factors included small Maf family members including Mafk, Mapff, and Mafg, all known heterodimeric binding partners with Nrf2.

### A rank-based strategy to predict Nrf2 activation

**Fig 3:**
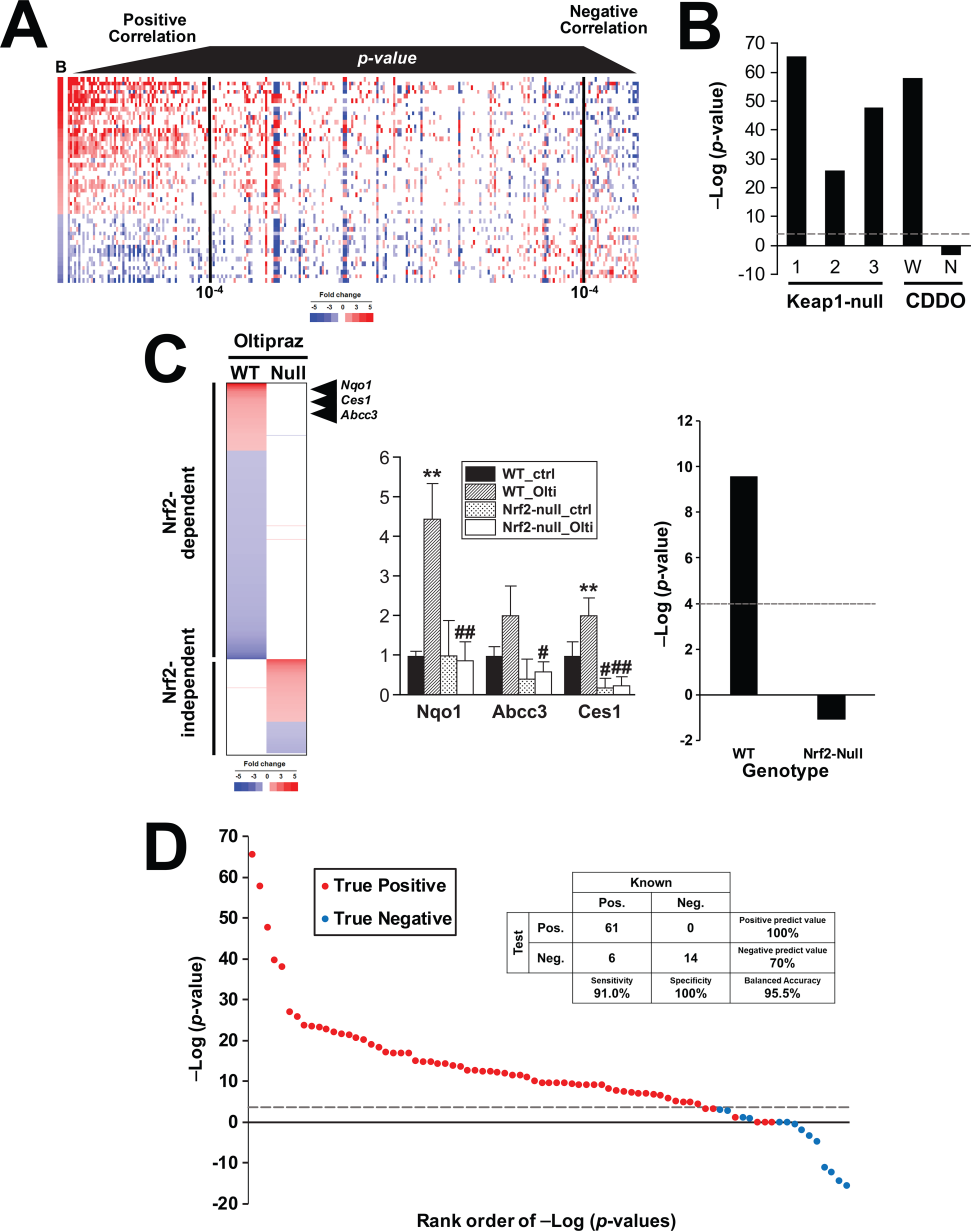
A rank-based strategy for prediction of Nrf2 modulation. A. Heat map showing the expression of genes in the Nrf2 biomarker across 443 biosets. Biosets were ordered based on their similarity to the Nrf2 biomarker using the p-value from the Running Fisher test. Biosets with positive correlation are on the left, and biosets with negative correlation are on the right. The black vertical lines denote a p-value of 10^−4^. B. The Nrf2 biomarker was compared to the biosets used to build the biomarker using the Running Fisher algorithm. Biosets with a negative correlation are shown as negative–log(p-value)s. C. Prediction of Nrf2 activation in the livers of mice exposed to oltipraz. Mice were exposed to oltipraz (4 days with 75 mg/kg/day), and the livers were examined for gene expression changes using Affymetrix mouse arrays. (Left) Heat map showing the expression of genes in wild-type and Nrf2-null mice. The genes were separated by strain-specific expression behavior and rank-ordered based on fold-change. The position of three genes in the Nrf2 biomarker are shown. (Middle) RT-PCR analysis of three biomarker genes in the livers of mice. Significantly different from corresponding control: * p < 0.05, **p < 0.01. Significantly different between controls in wild-type and nullizygous mice: ^#^ p < 0.05, ^##^ p < 0.01. (Right) The two biosets derived from the oltipraz treated vs. control comparisons in wild-type or Nrf2-null mice were evaluated for activation of Nrf2 using the Running Fisher test. D. Summary of the prediction sensitivity and specificity of the Nrf2 biomarker. The biomarker was compared to chemicals or other biosets that were known positives or negatives for Nrf2 activation. A total of 81 biosets from 32 studies were examined.

The biomarker was compared to statistically-filtered gene lists using the Running Fisher algorithm which computes a correlation direction (positive or negative) and an associated p-value of the correlation [44]. To visualize the relationship between the Running Fisher test p-value and the expression of genes in the biomarker, 443 biosets of statistically-filtered genes were evaluated for similarity to the Nrf2 biomarker and then sorted by p-value. Fig 3A (left side) shows that for biosets that had a positive correlation to the biomarker, the more significant the correlation (lower the p-value) the more the bioset exhibits similarities in expression of the biomarker genes in both direction and relative magnitude of the changes. These biosets included wild-type mice treated with CDDO-Im as well as comparisons between Keap1-disrupted mice vs. wild-type mice. Fig 3A (right side) shows a smaller group of biosets which exhibited negative correlation to the biomarker. The biosets on the farthest right exhibit the most significant negative correlation to the biomarker. In general, these biosets exhibited a pattern of expression that was opposite to that of the biomarker. Biosets in this group included comparisons between Nrf2-null mice vs. wild-type mice (discussed below).

The Nrf2 biomarker was evaluated for ability to correctly classify biosets with known Nrf2 activation status. Not surprisingly, the biosets from either the wild-type mice treated with CDDO-Im or the three Keap1-disrupted vs. wild-type comparisons exhibited statistically significant correlations (p-values ≤ 1E-26), whereas the bioset from CDDO-Im treated Nrf2-null mice did not have significant positive correlation (Fig 3B).

A number of studies have evaluated the transcriptional differences in chemically treated wild-type and Nrf2-null mice (e.g., [55]), but only one of these studies used multiple biological replicates allowing for a statistical analysis [47]. A separate study was carried out to independently evaluate the ability of the biomarker to predict Nrf2 activation. Wild-type and Nrf2-null mice were exposed to the known Nrf2 activator oltipraz as described in the Methods and livers were analyzed for gene expression by microarrays. A comparison of the genes altered by exposure showed that all but one of the 642 genes altered in wild-type mice was not altered by oltipraz in Nrf2-null mice (Fig 3C, left). Genes altered by oltipraz in wild-type but not Nrf2-null mice included *Nqo1*, *Ces1g* and *Abcc3* which are part of the Nrf2 biomarker. The expression of these genes was confirmed by RT-PCR (Fig 3C, middle). As shown in Fig 3C, right, oltipraz treatment in wild-type mice exhibited significant Nrf2 activation, while activation was abolished in the oltipraz-treated Nrf2-null mice. Thus, the biomarker accurately predicted the expected Nrf2 activation induced by oltipraz exposure in wild-type but not Nrf2-null mice.

To determine the predictive behavior of the biomarker, classification was performed on a set of biosets with known Nrf2 activation status. Criteria for classification included a p-value ≤ 1E-4. The final number of biosets evaluated were 67 positives and 14 negatives. Using this relatively modest number of biosets, the biomarker had a 91% sensitivity and a 100% specificity, giving a balanced accuracy of 96% (Fig 3D).

### The livers of female mice exhibit greater background Nrf2 activation than male mice

There are published examples of sex differences in response to various stressors that may induce Nrf2 [35]. The molecular basis of these responses is not clearly understood. Given the central role of the Nrf2-Keap1 regulon in protecting the cell from environmentally-induced oxidative stress, we hypothesized that Nrf2 activity contributes to sex differences in response to chemical-induced oxidative stress. To test this hypothesis, we determined whether differences in Nrf2-regulated genes exist between the sexes in mice. A large number of female vs. male comparisons (86) are found in the mouse liver compendium. The comparisons were divided into 4 groups based on the background factor that was tested in each of the experiments: 1) 40 control comparisons from untreated mice or mice treated with a control vehicle as part of toxicogenomics studies, 2) 19 comparisons in which mice were administered chemicals under identical conditions, 3) 15 comparisons in genetically altered mice, and 4) 12 comparisons in mice fed a high fat diet. Only sexually mature animals which exhibit robust sex differences in liver gene expression [34] were used in these comparisons.

**Fig 4:**
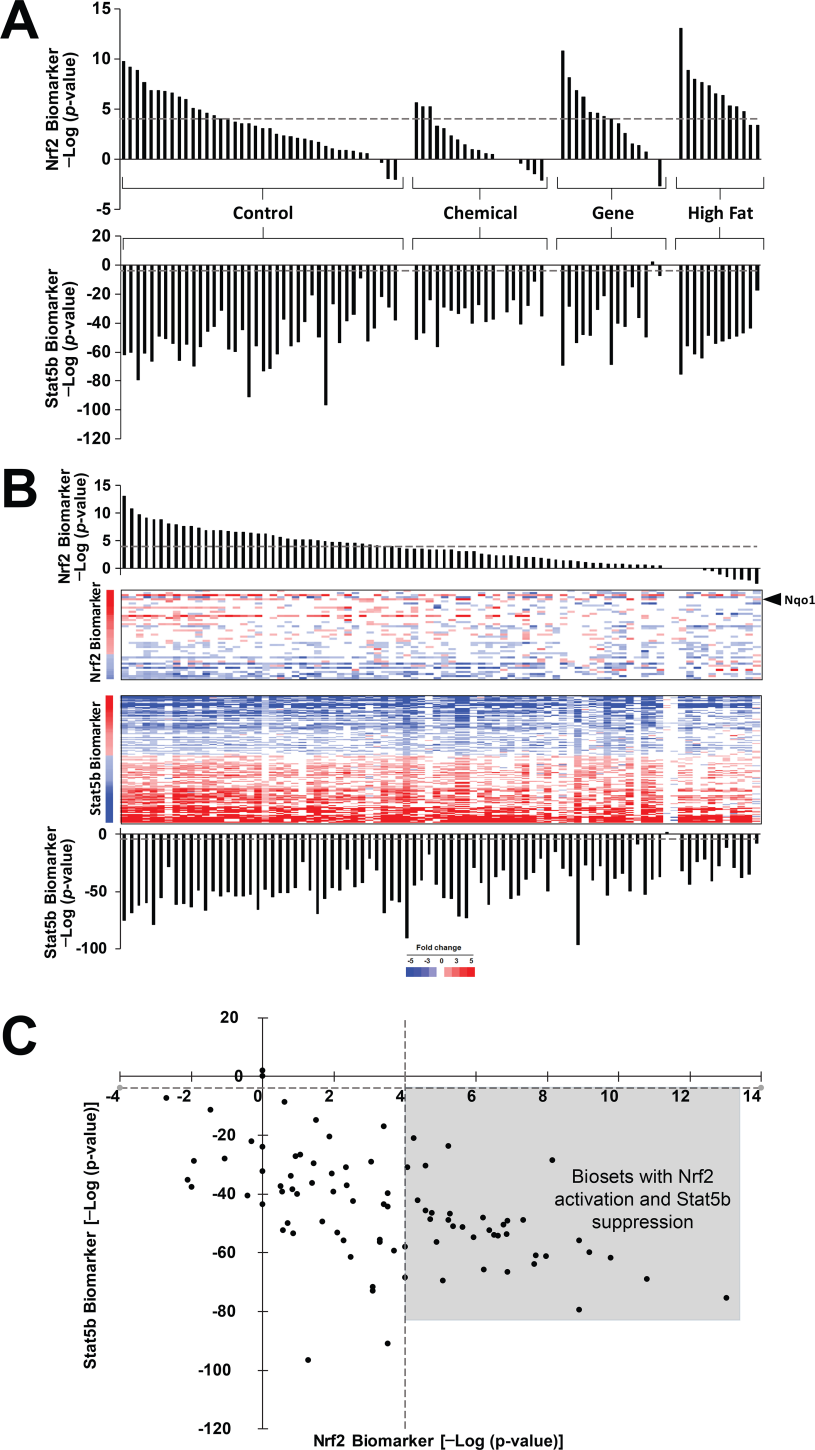
Activation of Nrf2 in females compared to males. A. Activation of Nrf2 in the livers of female mice compared to male mice. (Upper) Female vs. male comparisons were examined for effects on Nrf2. Biosets were divided into the indicated four groups based on underlying factors in common between the mice. Control biosets are from wild-type mice which in some studies include treatment with control vehicle. Only sexually mature mice were used in the analysis. (Lower) Each of the biosets were also compared to the biomarker for STAT5b [33]. The results show the expected suppression of the male-specific STAT5b gene expression pattern in most of the female mice compared to male mice. B. Expression of Nrf2 and STAT5b biomarker genes in female to male comparisons. The biosets of female vs. male comparisons from A. were rank-ordered based on the significance of the overlap with the Nrf2 biomarker. C. Relationship between Nrf2 activation and STAT5b suppression. Biosets from (A) were plotted based on the significance of the Nrf2 and STAT5b biomarker comparisons. The plot shows the trend toward more significant Nrf2 activation with more significant suppression of STAT5b. The points in the box are those female vs. male comparisons with significant activation of Nrf2 and significant suppression of STAT5b.

The biosets of female vs. male comparisons were analyzed for similarity to the Nrf2 biomarker using the Running Fisher algorithm as described in the Methods. Fig 4A (top) shows that 37 of the comparisons exhibited significant Nrf2 activation (p-value ≤ 1E-4), while most of the other comparisons exhibited a trend toward activation of Nrf2. None of the comparisons exhibited significant Nrf2 suppression. As all comparisons are organized as female vs. male, significant activation (p-value ≤ 1E-4) indicates a relative increase in Nrf2 activation in females compared to males. Suppression (p-value ≥ 1E-4), would indicate relative activation in males. Increased Nrf2 activation in females could result from true activation of Nrf2 in females, suppression of Nrf2 signaling in males, or a combination of both.

Our group had previously built and validated a gene expression biomarker that accurately predicted STAT5b activation or suppression (balanced accuracy = 97% or 99%, respectively; [33]. Using the STAT5b biomarker, the relationships between Nrf2 and STAT5b responses in these female vs. male comparisons were examined. Fig 4A (bottom) shows the expected suppression of the male-specific STAT5b activation (i.e., feminization) in most of the comparisons. Out of all of the biosets examined, only two did not reach significance. One of these biosets would not be expected to exhibit STAT5b effects, because the comparison came from mice lacking an active *Pou1fd1 (Pit1)* gene which controls sex-specific GH secretion from the anterior pituitary. There was no obvious explanation to why the other bioset (mice treated for 2 wk with 5000 ppm o-nitrotoluene) did not exhibit feminization. The relationship between the Nrf2 predictions and expression of the genes in the biomarker is shown in Fig 4B. The heatmap shows that for female vs. male comparisons with significant Nrf2 activation (Fig 4B, left), many of the genes exhibited expected behavior, including *Nqo1*. Genes in the Nrf2 biomarker that exhibited significant sex differences included those that had decreased expression in females compared to males (e.g., *Cyp4v3*, *C8a*, *Nudt7*, *Car3*, *Tsc22d1*). A plot of the–log(p-value)s of the Nrf2 and STAT5b predictions shows a trend toward more significant Nrf2 activation with more significant suppression of STAT5b (R^2^ = 0.163; p-value = 2.25E-4) (Fig 4C). All 37 biosets which exhibited significant Nrf2 activation also exhibited significant suppression of STAT5b, i.e., feminization (shaded area). In summary, the results demonstrate subtle but consistent sex differences in Nrf2 activation with females exhibiting greater Nrf2 activation than males under a number of diverse conditions.

### Hormonal regulation of Nrf2 activation

**Fig 5:**
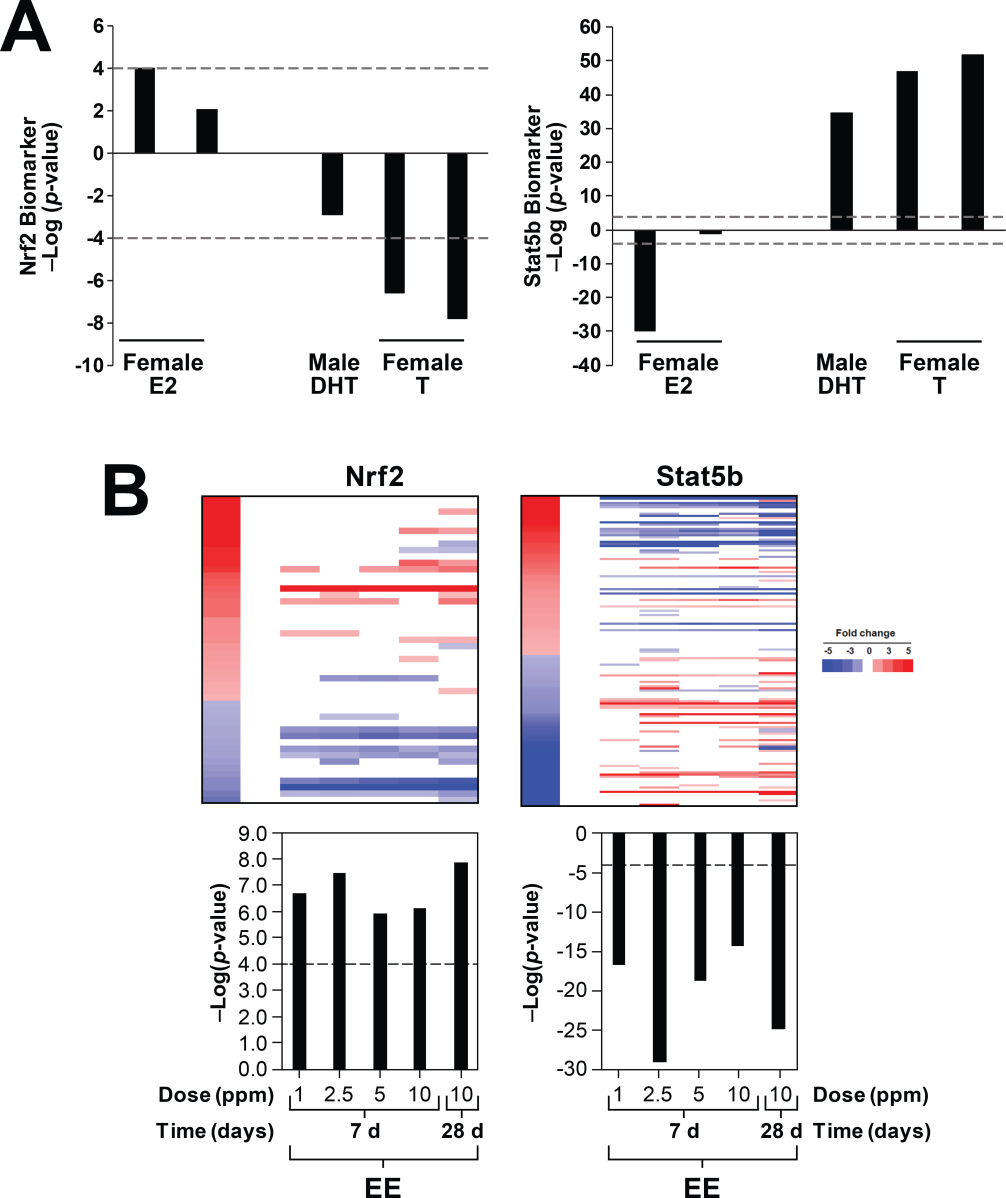
Nrf2 activation is hormonally-regulated. A. (left) Nrf2 activation is suppressed by testosterone and activated by estradiol. Effects on the Nrf2 biomarker were examined after treatment with estrogen, testosterone, or dihydrotestosterone in the indicated studies. Biosets were derived from GSE13265 and GSE13388. (Right) The same biosets were examined for effects on STAT5b. B. Nrf2 is activated by ethinyl estradiol in male mice. Mice were treated with ethinyl estradiol for 7 or 28 days at the indicated doses and then evaluated for effects on Nrf2 (left) or STAT5b (right). Biosets were derived from GSE84590.

Sex differences in the hepatic transcriptome are under control of sex hormones. It was hypothesized that Nrf2 activation is also under control of sex hormone levels. To test this hypothesis, biosets were evaluated in which sex hormone levels were altered; these included effects of castration, ovariectomy, and exogenous treatments with testosterone (T), dihydrotestosterone (DHT), or estradiol (E2) (from studies GSE21065, GSE13265, GSE13388). Ovariectomy compared to intact mice from two biosets had no effect on Nrf2 activation, and two biosets from castrated mice exhibited Nrf2 activation, but these comparisons did not reach statistical significance (p-value > 1E-2) (data not shown). Fig 5A (left) shows the effects of E2 (left) and T or DHT (right) treatment on Nrf2 activation. E2 treatment when compared to DHT treatment in ovariectomized females resulted in activation of Nrf2 that was marginally significant (p-value = 1E-4) (Lane 1). Ovariectomized mice administered E2 exhibited positive correlation but did not reach significance (Lane 2). Treatment of intact female mice with T or DHT resulted in negative correlation which for two biosets from mice treated with T for 3 wks caused a significant suppression of Nrf2 (Lanes 5 and 6). STAT5b activation assessed in the same biosets showed expected behavior including suppression by E2 and activation by DHT or T (Fig 5A, right).

We next determined the dose- and time-dependent effects of exposure to the estrogen receptor agonist ethinyl estradiol (EE). Mice were exposed for 7 or 28 days to EE in the feed (described in [56]). Nrf2 was significantly activated by EE at 7 and 28 days (Fig 5B, left). EE suppressed STAT5b, as expected (Fig 5B, right). Taken together, these studies indicate that estrogens increase Nrf2 activation and androgens suppress Nrf2 activation.

### Genetic models of aberrant growth hormone signaling exhibit coincident Nrf2 activation and feminization

**Fig 6:**
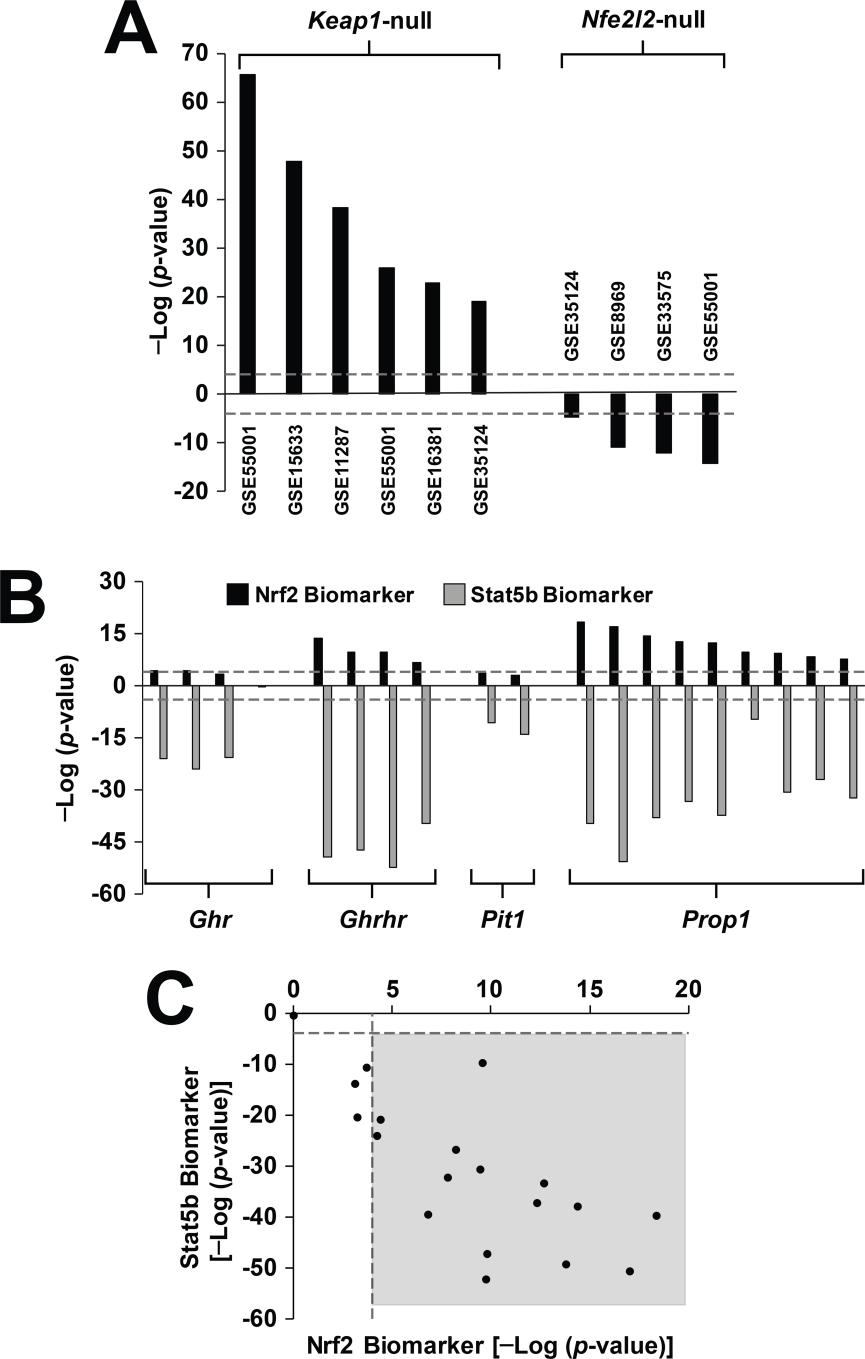
Parallel activation of Nrf2 and suppression of STAT5b in genetic models of disrupted growth hormone signaling. A. Expected Nrf2 biomarker behavior in biosets from Keap1-null or Nrf2-null mice. Biosets from nullizygous vs. wild-type comparisons from the indicated studies were assessed for Nrf2 activation. B. Nrf2 is activated in dwarf mice. Significance of the correlation of dwarf mouse vs. wild-type comparisons from the indicated mice to the Nrf2 or STAT5b biomarkers are shown. C. Relationship between activation of Nrf2 and suppression of STAT5b in the dwarf mouse comparisons from B. Points in the shaded box are those biosets in which there is significant Nrf2 activation and suppression of STAT5b.

Given the higher Nrf2 activation in females than males, the effects of genetic feminization on Nrf2 were examined. First, to ensure that the biomarker can accurately identify mutations that affect Nrf2 activity, we examined effects of nullizygous mutations in either Keap1 or Nrf2 on the Nrf2 biomarker predictions (Fig 6A). All of the Keap1-null biosets resulted in activation of Nrf2, and all of the Nrf2-null biosets resulted in suppression, as predicted. Mutations in genes that affect GH responsiveness and cause feminization were examined next to determine effects on Nrf2. Biosets were from mouse models with mutations that 1) abolish GH secretion because of defects in the differentiation of the anterior pituitary (*Pou1fd1 (Pit1)*, *Prop1*), 2) prevent the synthesis and secretion of GH from the pituitary (*Ghrhr*, *Ghrh*), or 3) affect the function of the GH receptor (*Ghr*). All of these mutations result in dwarfism and most lead to increases in longevity compared to wild-type mice through a delay in age-dependent diseases [35]. Biosets derived from comparisons between these mutant mice vs. their wild-type counterparts were examined for their effects on Nrf2 and STAT5b. Fig 6B (top) shows that most of these biosets resulted in significant activation of Nrf2 with the strongest effects found in *Ghrhr* and *Prop1* mutants. None of the *Pit1* mutant biosets reached significance. Biosets from *Ghr* deletion mutants exhibited different effects on Nrf2 depending on the location of the deletion. *Ghr* truncated at amino acid 569 (GSE11396, GSE988) had no effect on Nrf2 or STAT5b. These results were consistent with the weak to moderate effects on GH-dependent phosphorylation of STAT5b by JAK2 activated through GHR [57]. In contrast, truncation at amino acid 391 or deletion of the Box1 domain of GHR which binds JAK2 resulted in complete abolishment of phosphorylation of STAT5b [58] and in our study, resulted in significant Nrf2 activation and feminization. In addition, Nrf2 was activated in one bioset from *Ghrh*-null vs. wild-type mice (p-value = 2.2E-7; [38]). Similar to the analysis with the female vs. male comparisons, the significance of the suppression of STAT5b increases with the significance of the Nrf2 activation (R^2^ = 0.558; p-value = 2.4E-4) (Fig 6C). Taken together, the results generally show that disruption of male-specific GH secretion or signaling results in increases in Nrf2 activation.

### Feminization by chemical exposure is associated with Nrf2 activation

**Fig 7:**
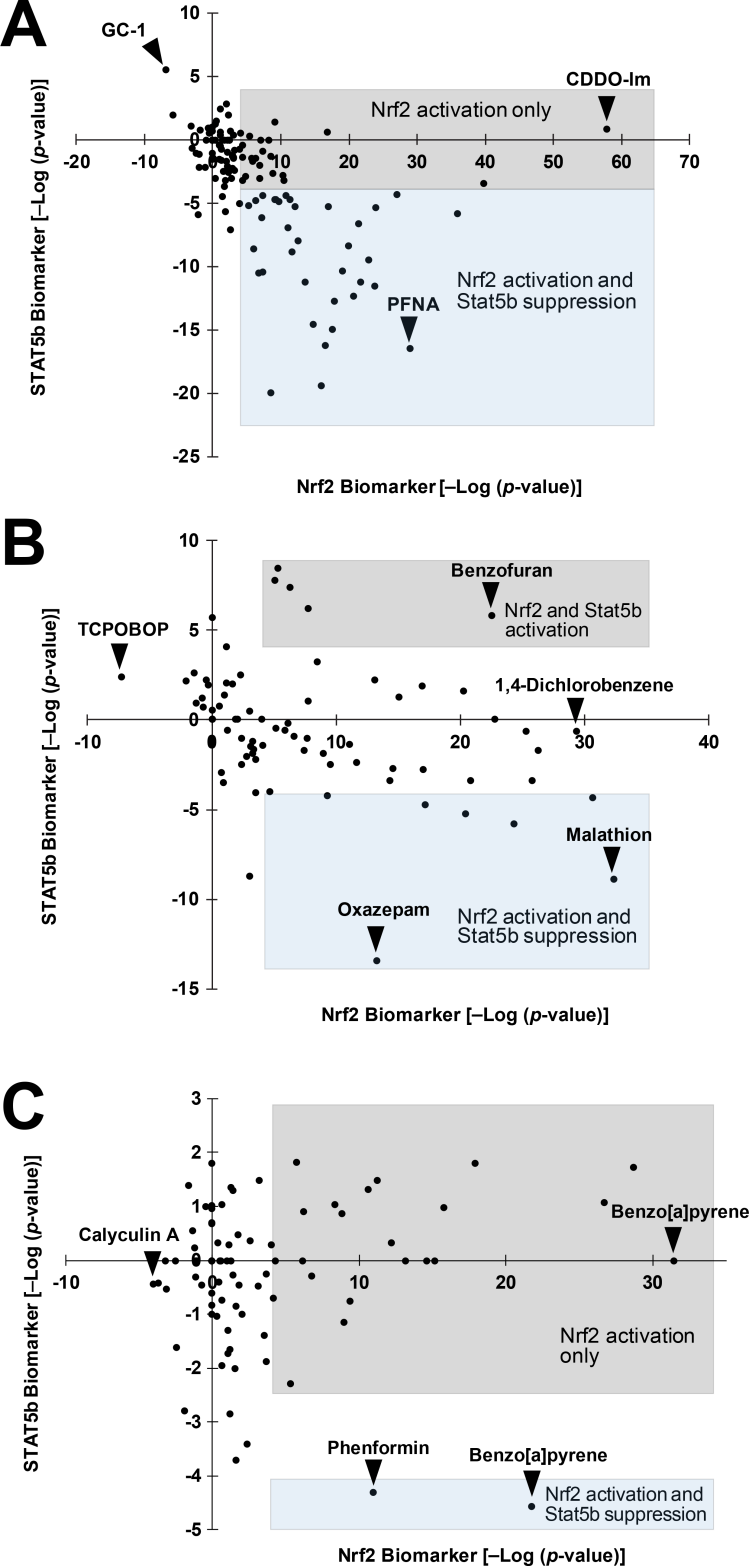
Feminization is associated with Nrf2 activation after chemical exposure in male mice. A. Coincidence of Nrf2 activation and STAT5b suppression after chemical exposure in male mice. Shaded boxes divide those biosets of significant Nrf2 activation into those with and without significant suppression of STAT5b. Specific chemicals mentioned in the text are shown. PFNA, perfluorononanoic acid; GC-1, a thyroid hormone receptor beta-specific agonist. B. Nrf2 activation and STAT5b effects in female mice. C. Uncoupling of Nrf2 activation and STAT5b suppression in chemically-treated primary hepatocytes. Biosets from chemically treated primary hepatocytes or hepatocyte-derived cell lines almost always exhibit Nrf2 activation in the absence of feminization. Two biosets showed activation of Nrf2 and suppression of STAT5b.

Feminization of the liver transcriptome is a common feature upon chemical treatment in male mice [33]. We hypothesized that chemically-induced feminization in male mice, like genetically-induced feminization, is associated with Nrf2 activation. In male mice, there was a striking relationship between feminization (STAT5b suppression) and Nrf2 activation (R^2^ = 0.170; p-value = 1.22E-6) (Fig 7A). Most (36 out of 59; 61%) of the biosets which exhibited significant Nrf2 activation also had significant coincident feminization (typified by perfluorononanoic acid (PFNA)). Chemicals that activated Nrf2 and caused feminization included benzo(a)pyrene, bezafibrate, ciprofibrate, galactosamine, oxazepam, perfluorohexane sulfonate, pregnenolone-16 alpha-carbonitrile, sebacic acid, T0901317, and WY-14,643. Most of these chemicals are activators of AhR, CAR and/or PPARα [39–41]. Biosets which lacked feminization generally exhibited less significant Nrf2 activation compared to those with coincident feminization. Chemicals that caused Nrf2 activation in the absence of feminization included acetaminophen, arochlor 1260, CDDO-Im, lipopolysaccharide, metformin, and triadimefon (**S1 File**). Only one bioset caused suppression of Nrf2 and STAT5b activation (GC-1, a thyroid hormone receptor activator).

In female mouse livers, there was no clear relationship between Nrf2 activation and feminization after chemical exposure (R^2^ = 0.108; p-value = 0.003) (Fig 7B). For most of the chemical treatments that activated Nrf2, there were few significant changes in STAT5b. Only 20% (8 of 40) of the biosets with activated Nrf2 showed coordinate suppression of STAT5b. Chemicals that activated Nrf2 in the absence of effects on STAT5b included 1,2,3-trichloropropane, 1,2-dibromoethane, 1,4-dichlorobenzene, 4-nitroanthranilic acid, ADBQ, arochlor 1260, ciprofibrate, CITCO, iodoform, naphthalene, PCB153, pentachloronitrobenzene, phenylhydrazine, tris(2,3-dibromopropyl)phosphate, and WY-14,643 (**S1 File**). Chemicals that activated Nrf2 and caused feminization included 1,5-naphthalenediamine, malathion, oxazepam, phenobarbital, propylene glycol mono-t-butyl ether, TCDD, and TCPOBOP. Benzofuran, coumarin, and methylene chloride caused increases in Nrf2 activation and activation of STAT5b (masculinization). TCPOBOP was the only chemical that caused suppression of Nrf2 with no effects on STAT5b. Overall, these results indicate that coincident activation of Nrf2 and suppression of STAT5b often occurs in male but not female mice.

### Uncoupling of chemical-induced Nrf2 activation and STAT5b effects in vitro

We hypothesized that if the linkage between Nrf2 and STAT5b is hormonally-driven, the observed coupling of effects would be absent in in vitro cultures. To test this hypothesis, biosets from chemically-treated primary hepatocytes or hepatocyte cell lines were examined. Fig 7C shows that while many of the treatments resulted in activation of Nrf2, only 2 of the biosets exhibited suppression of STAT5b (R^2^ = 0.009; p-value = 0.400). The chemicals that altered Nrf2 in vitro in the absence of effects on STAT5b are summarized in S1 File and included 2-(chloromethyl)pyridine.HCl, 2-acetylaminofluorene, 2-NP, curcumin, diethyl maleate, dimethylbenzanthracene, ethanol, lead acetate, o-anthranilic acid, quercetin, and sodium arsenite. Benzo[a]pyrene exposure (5uM for 24h) led to the greatest Nrf2 activation but no STAT5b effect (from E-TABM-1139). The two chemicals that activated Nrf2 and caused weak STAT5b suppression included phenformin and benzo[a]pyrene (30uM for 36h from E-MEXP-2209/2539). Calyculin A was the only chemical that caused Nrf2 suppression. The lack of linkage between Nrf2 activation and feminization in in vitro cultures indicates that the coupled effects require an in vivo environment of hormonal disruption that is not recapitulated in vitro.

### Females exhibit greater chemical-induced Nrf2 activation than males

**Fig 8:**
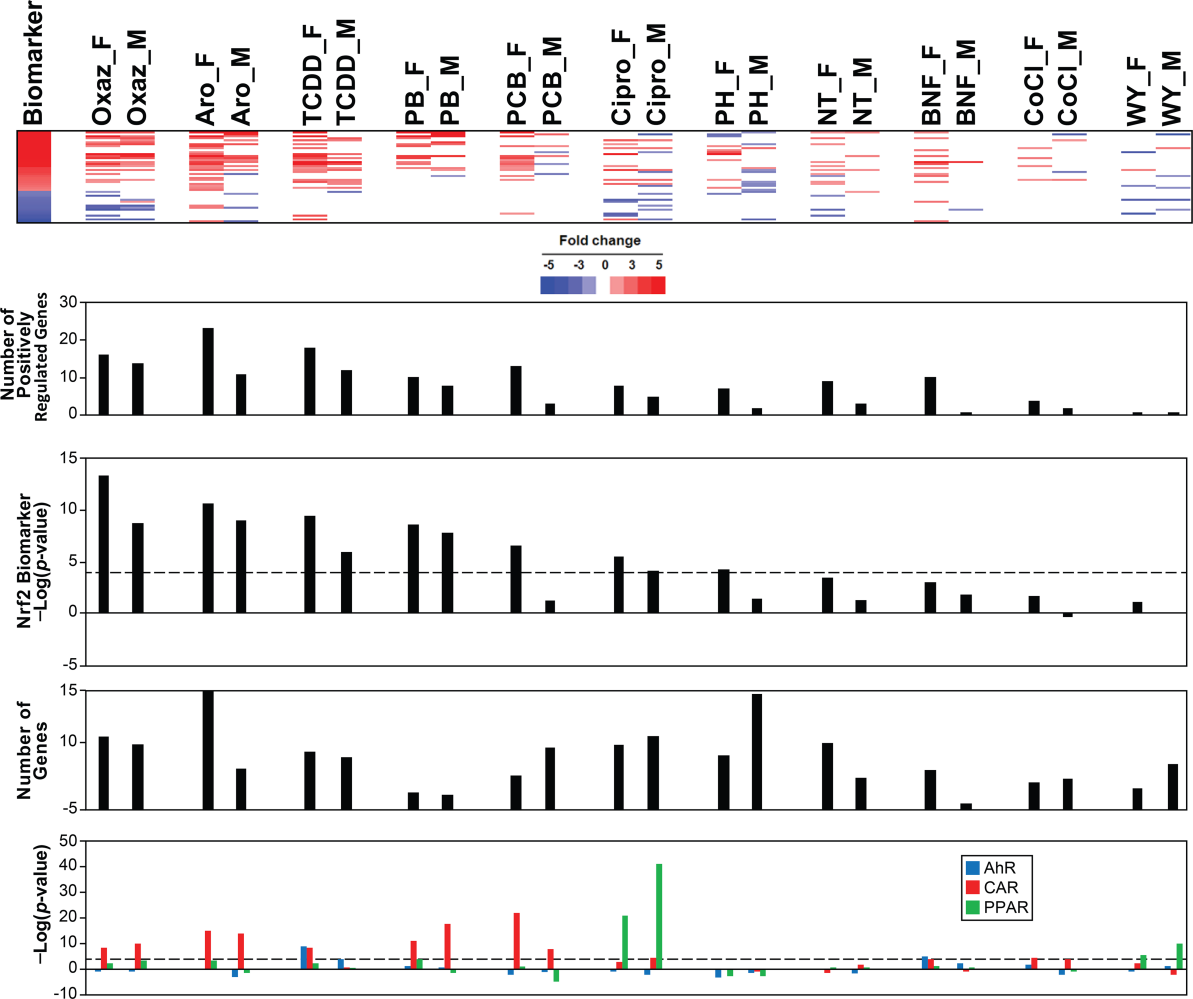
Greater numbers of Nrf2 biomarker genes are activated in females than males after chemical exposure. Male and female mice were exposed to the indicated chemicals as described in the Methods and Table 1 and assessed for Nrf2 activation. From top to bottom panels: expression of Nrf2 biomarker genes, the number of positively regulated Nrf2 biomarker genes altered, the -log(p-value)s of the correlation of the bioset to the Nrf2 biomarker, total number of genes altered by the chemical treatment, and -log(p-value)s of the correlation to the indicated biomarkers for xenobiotic receptors.

Given the higher basal activation of Nrf2 in females than males, we hypothesized that there would be sex differences in Nrf2 responsiveness after chemical exposure. Male and female mice were exposed under identical conditions to 11 chemicals (Table 1). The livers of the mice were examined by full genome transcript profiling and examined for Nrf2 effects using the biomarker. Remarkably, in all chemical treatments, the females exhibited more significant Nrf2 activation reflected in a greater number of significantly altered positively regulated Nrf2 biomarker genes in female livers compared to male livers (Fig 8). Sex differences in responses were notable for Aroclor, TCDD, PCB-153, and phenylhydrazine. The activation of AhR, CAR and PPARα was examined using characterized biomarkers [39–41]. While the majority of the comparisons did not have major differences in correlation to the biomarkers between sexes, TCDD and PCB153 exhibited greater correlation to the AhR or CAR biomarkers, respectively in females compared to males.

**Table 1:** Exposure conditions of 11 chemicals evaluated for sex differences in Nrf2 activation.

Chemical	Abbreviation	Dose or Concentration	Duration	Route	Study
**2,3,7,8-Tetrachlorobenzodioxin**	TCDD	10 ug/kg	2 days	Injection	GSE55084
**Aroclor 1620**	Aro	200 mg/kg	2 days	Injection	GSE55084
**Beta-naphthoflavone**	BNF	50 mg/kg	1 day	Injection	GSE55084
**Ciprofibrate**	Cipro	250 mg/kg	8 hrs	Gavage	GSE55084
**Cobaltous chloride**	CoCl	60 mg/kg	2 days	Injection	GSE55084
**o-Nitrotoluene**	NT	5000 ppm	2 wks	Feed	E-TOXM-18
**Oxazepam**	Oxaz	2500 ppm	2 wks	Feed	E-TOXM-18
**PCB153**	PCB	80 mg/kg	2 days	Injection	GSE55084
**Phenobarbital**	PB	100 mg/kg	3 days	Injection	GSE55084
**Phenylhydrazine**	PH	100 mg/kg	2 days	Injection	GSE55084
**WY-14,643**	WY	250 mg/kg	8 hrs	Gavage	GSE55084

Sex differences could be due to differences in levels of internal exposure to the compounds which could lead to sex differences in total number of genes altered. However, there was no clear relationship between higher Nrf2 activation and number of genes altered by the chemical in female mice. For the chemicals with the greatest sex differences, the number of genes that were differentially expressed in the livers was either about the same in each sex (1305 vs. 1472 for males and females treated with TCDD) or greater in males than females (1567 vs. 870 or 2901 vs. 1353 for PCB-153 and phenylhydrazine, respectively). These results appear to identify a set of chemicals that induce greater Nrf2 activation in females compared to males.

## Discussion

Annotated databases of microarray data provide opportunities through novel methods of reanalysis to gain insights into the network of factors altered by chemical exposure. In the present study, we used a gene expression biomarker that can accurately predict the activation status of the oxidant-induced transcription factor Nrf2 in a microarray database and uncovered a strong linkage between increased Nrf2 activity and feminization of the male liver mediated by suppression of STAT5b. Examination of female versus male comparisons showed that Nrf2 is subtly but reproducibly activated to higher levels in females. Additional analyses showed that feminization of the liver transcriptome by genetic, dietary or chemical modulation under conditions which suppressed STAT5b consistently led to Nrf2 activation. Finally, we found that female mice generally exhibited greater induction of Nrf2-regulated genes after chemical exposure compared to male mice. These studies indicate that feminization of the liver transcriptome through many stressors leads to increased Nrf2 activity. The higher basal and chemical-induced Nrf2 activity may explain in part why female mice are often more resistant to a number of stressors compared to males.

The Nrf2 biomarker gene set comprised of 52 genes was built using liver microarray profiles in which Nrf2 was activated pharmacologically by CDDO-Im or genetically in Keap1-disrupted mice. These Nrf2-dependent genes included the prototypical targets of Nrf2 including *Nqo1* and *Gst* family members (Fig 2A). IPA upstream activator function ranked Nrf2 as the most likely transcription factor for regulating the biomarker genes (Fig 2C). The gene biomarker reliably predicted Nrf2 activation, with a balanced accuracy of 96% (Fig 3D). This high degree of accuracy was similar to predictions of the activation of other transcription factors in the mouse liver or human cell lines assessed using a similar approach [33, 34, 39–42]. The methods used including a combination of expert-generated gene biomarkers and the Running Fisher algorithm is a useful strategy to assess modulation of transcription factors in compendia of gene expression profiles.

To screen for factors in the microarray database that lead to alterations of Nrf2 function, we compared the Nrf2 biomarker to a gene expression compendium of ~2500 annotated microarray comparisons allowing an unprecedented assessment of factors that control Nrf2 activity. The compendium contains 86 female vs. male biosets in which female and male mice were evaluated under identical experimental conditions (Fig 4A). Almost all of the female vs. male biosets exhibited positive correlation with the Nrf2 biomarker (Fig 4B). The sexual dimorphic nature of Nrf2 activation is strongly supported by a study examining the circadian expression of Nrf2 or target genes. *Nfe2l2*, *Keap1*, *Nqo1*, *Gpx1*, and *Gst* family members exhibited higher gene expression in females than males that was dependent on the time of day the expression was measured [59]. Females exhibited greater expression of the Nrf2-regulated genes and lower reduced glutathione levels than males during the light period between 6:00 and 18:00. Only *Nqo1* was consistently elevated in females compared to males throughout the 24 hr day. The sexual dimorphism of hepatic gene expression is also under circadian clock control, as *Cry*-/- mice are unable to synthesize CRY1 and CRY2 proteins, have altered growth hormone (GH) levels and cannot sustain hepatic sex-differences in some drug metabolic genes [60]. The fact that there appeared to be differences in the level of Nrf2 activation between females and males in our comparisons in the compendium could be due to the different times of the day the animals were sacrificed. In addition, variability may come from chemical, genetic, or high fat diet perturbation of the circadian rhythmicity of sexual dimorphism. The large number of female versus male comparisons in the compendium exhibiting small but consistent Nrf2 alterations allowed greater confidence in our conclusion that females exhibit higher Nrf2 activity than males.

Based on the sexual dimorphism in Nrf2 regulation and the fact that liver sexual dimorphism is under control of STAT5b, we hypothesized that hormone, genetic, dietary, and chemical treatments that disrupt the GH-regulated liver transcriptome dependent on STAT5b would lead to Nrf2 activation. Hormone treatments that activate STAT5b (testosterone or dihydrotestosterone) caused suppression of Nrf2, while treatments that suppress STAT5b (estrogen, ethinyl estradiol) caused activation of Nrf2 (Fig 5A and 5B). Very little data exists that provides direct evidence of hormonal regulation of Nrf2. Maternal exposure to two compounds in mice that can activate estrogen receptor (genistein and quercetin) led to increased expression of *Nfe2l2* and *Hmox1* genes and decreased ROS-induced DNA damage as measured by 8-Oxo-dG and M1dG adducts in the livers of 12-wk old male offspring [61]. Dietary supplementation with genistein in rats led to increased expression of liver *Nqo1* [62]. There were no measurements of markers of GH-regulated genes in these studies. Nrf2 was found to directly regulate estrogen sulfotransferase (*Sult1e1*) which sulfonates and deactivates estrogens [14]. In a model of ischemia and reperfusion injury (I/R), the authors found that Nrf2 activation could be augmented by ablation of EST and suppressed by ovariectomy indicating that estrogens activate Nrf2. Whether the mechanism for estrogen activation of Nrf2 is direct or indirect has not been determined. It should be noted that in some in vitro settings the estrogen receptor signaling pathway has an inhibitory effect on Nrf2-dependent responses as antiestrogens upregulate Nrf2 signaling [63–65].

There was also dietary and genetic evidence for coupling of Nrf2 activation and STAT5b suppression. Fasting for up to 48 hrs is known to disrupt GH signaling at a number of nodes in the hypothalamic-pituitary-liver axis [66]. Most of the 10 biosets in the compendium in which mice were fasted exhibited Nrf2 activation (data not shown). A number of mouse models with disrupted GH signaling exhibited consistent activation of Nrf2 in all biosets derived from mice with mutations in *Prop1*, *Ghrhr*, and *Ghrh* (Fig 6B). There was weak activation in the biosets from mice with a mutation in *Pou1f1*. Mice with mutations in the GH receptor that abolished GH effects also exhibited Nrf2 activation. Our findings are consistent with a growing body of evidence that the Nrf2 pathway is activated in the livers of mice with defects in GH signaling. Fibroblasts isolated from Snell *Pit1* dwarfs have elevated mRNA levels for several Nrf2-dependent genes, as well as cellular traits, such as elevated glutathione levels, resistance to lipid peroxidation, that are known to be modulated by Nrf2 function [36]. A set of Nrf2-dependent mRNAs was elevated in the livers from young adult Ames *Prop1* and Snell dwarf mice in the absence of chemical induction [36, 37]. More recently, *Ghrh*-null mice were shown to have higher nuclear accumulation of Nrf2 protein in hepatocytes compared to wild-type mice and express higher levels of Nrf2 targets [38]. Our study is the first to show that with the exclusion of the *Pit1* mice, dwarf mice consistently exhibit liver Nrf2 activation. Overall, the data supports the relationship between genetic disruption of the GH hypothalamic-pituitary-liver axis and Nrf2 activation.

We found that female mice appear to mount a greater Nrf2 response to chemical exposure than male mice. After exposure to 11 chemicals identically administered to male and female mice, Nrf2 was activated to a higher level in female mice than male mice (Fig 8) based on the greater number of biomarker genes positively regulated by Nrf2. The greater induction of Nrf2 in females was not due to greater numbers of all genes perturbed by the chemical treatment compared to males. Out of the 11 chemicals about half (6) had higher numbers of significantly altered genes in females than in males. There is some evidence that a higher background of Nrf2 activation in different genetic and sex contexts can lead to a greater Nrf2 response once challenged with a chemical. In Pit1 Ames dwarf mice, which had higher basal level of Nrf2 activation compared to wild-type counterparts, there was a greater induction of Nrf2-regulated genes (*Hmox1*, *Nqo1*, *Mt1*, *Mt2*) after exposure to the oxidative stress inducer diquat [37]. Exposure to 4-aminobiphenyl in wild-type mice led to greater elevation of liver nuclear Nrf2 protein and expression of Nrf2 targets, *Ggt1* and *Nqo1* in females compared to males [67]. There was higher Nrf2 target gene induction by acetaminophen at 4 and 24 hrs in females especially *Nqo1* and *Hmox1* genes as well as protein levels of Nqo1 and Gclc while there was less tissue damage in female mice [68]. Induction of *Nqo1* was found to be greater in females than males after exposure to TCDD [69]. Upon TCDD exposure female mice are less sensitive than males and develop fewer toxicities [70]. The differential response is caused, at least in part, by sex hormones; ovariectomized mice were more sensitive while castrated mice were more resistant to TCDD-induced lethality than intact mice [71]. These studies support a model in which female mice can mount a greater Nrf2 response to chemical exposure which may be due to the higher background levels of Nrf2 activation.

There are numerous studies which show a relationship between the activation status of Nrf2, resistance to various stressors, and longevity. Female mice live longer than males [72], possibly due to a higher defense mechanism against ROS [73]. Overexpression of the Nrf2 ortholog SKN-1 can prolong lifespan in *Caenorhabditis elegans* [74]. Disruption of *Keap1* in male *Drosophila melanogaster* increases Nrf2 activity and longevity [75]. In a comprehensive study examining the role of Nrf2 in mediating the beneficial effects of caloric restriction (CR), Nrf2 was required for CR to suppress liver tumors but not required for CR-induced longevity [76]. *Gsta4*-null mice, in which detoxification of the lipid peroxidation product 4-hydroxynonenal is impaired, exhibit Nrf2 activation and have an extended life span [77]. In a comparative approach examining Nrf2-signaling activity in the long-lived naked mole-rat and nine other rodent species with varying maximum lifespan potential (MLSP), constitutive Nrf2 activity was positively correlated with MLSP while species longevity was not linked to the levels of Nrf2 protein itself [78]. The dwarf mice examined above exhibit increased resistance to chemical stress, decreased cancer incidence and increased life-span [35]. More recently, male mice exhibit longer lifespans if treated with a weakly estrogenic agonist or a Nrf2 inducer [79]. When provided as a concentrated broccoli sprout extract, the Nrf2 activator sulforaphane reduced fasting blood glucose and glycated hemoglobin (HbA1c) in obese patients with dysregulated type 2 diabetes [80].

There is evidence for at least four nonexclusive mechanisms that link suppression of STAT5b and Nrf2 activation. In the first mechanism, estrogens can be metabolized by Cyp family members to 2- and 4-hydroxyestrogens which can activate Nrf2 [81] and suppress STAT5b presumably acting at the level of the hypothalamus to disrupt the male-specific pulsatile GH secretion pattern [33]. In the second model, Nrf2 is directly or indirectly activated through kinase cascades. These cascades include those regulated by GH involving phosphatidylinositol-3 kinase (PI3K), Akt, protein kinase C (PKC), and mitogen-activated protein kinase (MAPK) [82] that also activate Nrf2 [83]. In the third mechanism, the stress of chemical exposure leads to increased circulating glucocorticoids which can negatively affect GH signaling [84]. In the last mechanism, increased oxidative stress secondary to increased expression and activity of Cyp family members leads to activation of Nrf2 and suppression of STAT5b. We recently found that chemicals that activated the transcription factor CAR also activate Nrf2 and this may be due to increased activity of Cyp2b family members (Rooney et al., unpublished). Further work is needed to determine how the activities of Nrf2 and STAT5b are functionally linked.

## Supporting information

S1 File Biomarker, bioset and ChIP-seq information. Contains 1) list of probesets and genes in the Nrf2 biomarker, 2) description of biosets from chemical treatment experiments that activate or suppress Nrf2 and STAT5b discussed in this study, and 3) genes that are mapped nearest to the location of Nrf2 binding peaks from 4 published mouse ChIP-seq datasets.
(XLSX)

S2 File Classification analysis. Contains the section “Classification analysis of Nrf2 activation using machine learning algorithms”.
(PDF)

## Abbreviations

AhR: aryl hydrocarbon receptor
AOP: adverse outcome pathway
CAR: constitutive activated receptor
ChIP: chromatin immunoprecipitation
Ct: cycle threshold
Nrf2: nuclear factor erythroid-2-related factor 2
PB: phenobarbital
PCR: polymerase chain reaction
PPAR: peroxisome proliferator-activated receptor
STAT5b: signal transducer and activator of transcription 5b
TCDD: 2,3,7,8-tetrachlorodibenzo-*p*-dioxin
WY: WY-14,643
