# Supplementary material for: Activation of Nrf2 in the liver is associated with stress resistance mediated by suppression of the growth hormone-regulated STAT5b transcription factor
Source: PLoS One. 2018 Aug 16;13(8):e0200004. doi: 10.1371/journal.pone.0200004 (PMC6095522; doi:10.1371/journal.pone.0200004)
Supplement: S2 File — Contains the section “Classification analysis of Nrf2 activation using machine learning algorithms”. (PDF) [file pone.0200004.s002.pdf]

Supplemental File 2

Activation of Nrf2 in the Liver is Associated with Stress Resistance Mediated by Suppression of the Growth Hormone-Regulated STAT5b Transcription Factor

John Rooney, Keiyu Oshida[1, 2], Naresh Vasani[1], Beena Vallanat[1], Natalia Ryan[1], Brian N. Chorley[1], Xuting Wang[3], Douglas A. Bell[3], Kai C. Wu[4], Lauren M. Aleksunes[5], Curtis D. Klaassen[6], Thomas W. Kensler[7], and J. Christopher Corton[1,8]

[1]NHEERL, US-EPA, Research Triangle Park, NC 27711
[2]Present address: Toray Industries, Inc., Kanagawa, Japan
[3] Immunity, Inflammation, and Disease Laboratory, National Institute of Environmental Health Sciences, Research Triangle Park, NC 27709
[4]University of Kansas Medical Center, Kansas City, KS 66160Present address: Janssen Pharmaceuticals, Spring House, PA 19477
[5] Rutgers University, Ernest Mario School of Pharmacy, Department of Pharmacology and Toxicology, Piscataway, NJ 08854
[6]University of Washington, Seattle, WA 98195
[7] Department of Pharmacology & Chemical Biology, School of Medicine, University of Pittsburgh, Pittsburgh, Pennsylvania 15261 Department of Pharmacology and Molecular Sciences, School of Medicine, The Johns Hopkins University, Baltimore, Maryland 21205

Classification analysis of Nrf2 activation using machine learning algorithms.

Nrf2 activation was predicted using 7 classification models as detailed in the Methods section. To determine the pcontribution of comparisons between chemically-treated wild-type and Nrf2-null samples, two training sets were used in the prediction models including the samples from livers of wild-type and Nrf2-null mice treated with CDDO-Im (Yates et al., 2009) and the same dataset excluding the control and treated Nrf2-null samples. The derived classifiers of 175 and 92 probe sets, respectively were then used to predict Nrf2 activation of test samples. An independent manually curated test set came from mice with known Nrf2 activation status. The models using the wild-type and Nrf2-null samples in the training set had excellent sensitivity (mean, 100%) but low specificity (specificity range, 30-94%; mean, 62%) (data not shown). The models using only the wild-type samples as the training set had lower sensitivity compared to using all samples (mean, 57%) but somewhat greater specificity (range, 71-84%; mean, 78%), indicating that the wild-type vs. null comparison of CDDO-Im treatment contributed to improved sensitivity in classification predictions. Because of the low specificity or sensitivity of the models, none were thought to be adequate for predicting Nrf2 activation of additional samples.

Reference

Yates MS, Tran QT, Dolan PM, Osburn WO, Shin S, McCulloch CC, Silkworth JB, Taguchi K, Yamamoto M, Williams CR, Liby KT, Sporn MB, Sutter TR, Kensler TW. Genetic versus chemoprotective activation of Nrf2 signaling: overlapping yet distinct gene expression profiles between Keap1 knockout and triterpenoid-treated mice. Carcinogenesis. 2009 Jun;30(6):1024-31.
